# ONCOPLEX: an oncology-inspired hypergraph model integrating diverse biological knowledge for cancer driver gene prediction

**DOI:** 10.1038/s41598-026-36127-8

**Published:** 2026-01-13

**Authors:** Etab Mohammed Alotaibi, Omer S. Alkhnbashi, Van Dinh Tran

**Affiliations:** 1 Information and Computer Science Department, King Fahd University of Petroleum and Minerals (KFUPM), Dhahran, Saudi Arabia; 2 Center for Applied and Translational Genomics (CATG), Mohammed Bin Rashid University of Medicine and Health Sciences (MBRU), Dubai Health, Dubai, United Arab Emirates; 3 College of Medicine, Mohammed Bin Rashid University of Medicine and Health Sciences (MBRU), Dubai Health, Dubai, United Arab Emirates

**Keywords:** Cancer driver gene prediction, Hypergraph neural networks, Multi-omics integration, Cancer, Computational biology and bioinformatics

## Abstract

Cancer development is driven by a small subset of somatic mutations, known as driver mutations, that disrupt key regulatory processes in cells. These mutations occur in specific genes, called cancer driver genes, whose altered functions promote tumor initiation and progression. Accurately identifying driver genes remains a major challenge due to their rarity and the overwhelming presence of passenger mutations. Recent advances in graph-based deep learning have improved the modeling of gene interactions, but most approaches are limited to pairwise connections and fail to capture the higher-order complexity of biological systems. We introduce ONCOPLEX, a hypergraph-based neural network framework that models genes as nodes and curated cancer-related pathways as hyperedges, enabling the representation of multi-gene interactions. Unlike previous methods, ONCOPLEX integrates diverse molecular and phenotypic features, such as somatic mutations, gene expression, and DNA methylation, into a pathway-informed hypergraph structure to learn biologically meaningful gene representations. ONCOPLEX is trained in a supervised manner on labeled driver and non-driver genes, with unlabeled genes included as nodes during representation learning. Comprehensive evaluations across pan-cancer and cancer-type-specific settings show that ONCOPLEX consistently outperforms state-of-the-art methods in classification and ranking metrics. It accurately recovers known driver genes and highlights novel candidates supported by literature and enrichment analyses. These findings underscore the power of pathway-guided hypergraph modeling for advancing cancer driver gene discovery.

## Introduction

Among the leading causes of death worldwide, cancer imposes a considerable burden on patients, healthcare systems, and society^1^. At its core, cancer arises from the accumulation of somatic mutations^2,3^. These mutations occur in a limited number of genes known as driver genes, which play an essential role in the regulation of key biological processes such as cell growth, cell cycle, and DNA replication, which ultimately contribute to cancer progression^4^. Identifying driver genes is a critical focus in cancer genomics, serving as the foundation for accurate cancer diagnosis and the development of personalized treatments^5^. However, this remains a major challenge due to the difficulty in distinguishing true driver mutations from passenger mutations, which do not directly contribute to disease progression. During the past decade, numerous computational methods have been proposed to address this challenge, each based on different biological assumptions and data modalities.

Mutation frequency-based approaches, for example, assume that driver gene mutations occur significantly more frequently than mutations in non-driver genes^6^. However, these methods are prone to false positives and often miss low-frequency driver genes. Recognizing that mutations in driver genes can affect other genes through biological interactions, network-based methods have emerged to improve prediction by propagating mutational signals through biological networks, incorporating both mutation data and network topology^7–10^. Traditional protein–protein interaction (PPI) networks provide the backbone for many of these methods, but are limited by incomplete or noisy interaction data.

To address these limitations, deep learning and machine learning techniques have been applied to learn complex mutational patterns from multi-omics data, including single-nucleotide variants (SNVs), copy number variations (CNVs), gene expression, and DNA methylation^11–13^. Although these methods offer improvements over frequency-based approaches, they still struggle to capture the intricate biological relationships underlying cancer effectively.

Graph Neural Networks (GNNs) have recently emerged as powerful tools that unify network-based and machine learning approaches. They enable the integration of diverse data modalities, such as multi-omics profiles, functional annotations, and clinical properties as node attributes in biological networks. For example, EMOGI^14^ used graph convolutional networks (GCNs) to learn gene representations from PPI networks enriched with some multiomics data, although its ability to identify novel driver genes remained limited. MTGCN^15^ introduced a multitask learning framework that combines node classification and link prediction, resulting in more informative embeddings. However, GNN-based approaches typically rely on the assumption of homophily, the idea that connected nodes tend to share similar labels. This assumption often fails in cancer biology, where important interactions may occur between functionally distinct or distant genes. This mismatch reduces the effectiveness of homophily-based models. HGDC^16^ proposed a diffusion-based method to construct auxiliary networks that better capture biological structure and mitigate over-smoothing, leading to improved driver gene prediction. However, most graph-based methods only model pairwise interactions, which limits their ability to capture higher-order phenomena, such as pathways or protein complexes. Reducing these complex relationships to binary edges overlooks the multifaceted nature of biological systems.

Hypergraphs extend traditional graphs by allowing hyperedges to connect multiple nodes simultaneously, enabling the modeling of higher-order relationships in biological processes. Several hypergraph-based approaches have shown promise in driver gene discovery. DriverRWH^17^, for example, applies random walks with restart on mutation-based hypergraphs, where each patient is represented as a hyperedge linking their mutated genes, capturing richer mutational contexts and reducing false positives. Similarly, personalized mutational hypergraphs have been shown to improve predictions in cancer genomics^18^. More recently, PITCH^19^ constructed personalized hypergraphs from pathway and PPI data to classify common and patient-specific driver genes, highlighting the benefit of integrating pathway-informed structures. DISHyper^20^ further advanced this direction by introducing a hypergraph neural network (HGNN) that forms hyperedges from curated biological annotations, allowing for the learning of gene representation within functional modules. However, DISHyper’s node features are derived solely from the hypergraph structure, based on memberships in the gene set, rather than molecular or phenotypic characteristics. Although these methods offer useful information, they are still limited in integrating heterogeneous data sources and capturing the complex interplay between node features and hypergraph topology.

We build on the assumption that integrating intensive and diverse biological characteristics as node features within a hypergraph whose topology is derived from curated, cancer-associated pathways offers a more biologically grounded and effective framework for cancer driver gene discovery. To this end, we propose ONCOPLEX, an oncology-inspired hypergraph-based model for cancer driver prediction. Unlike previous hypergraph models, ONCOPLEX combines a pathway-informed graph structure with rich, multi-source node features, enabling the learning of expressive gene representations. We perform comprehensive evaluations on pan-cancer and cancer-specific datasets, in which ONCOPLEX is compared with state-of-the-art methods. The results consistently demonstrate that our proposed method outperforms existing approaches in predictive accuracy and in identifying biologically relevant cancer drivers.

## Results

**Fig. 1:**
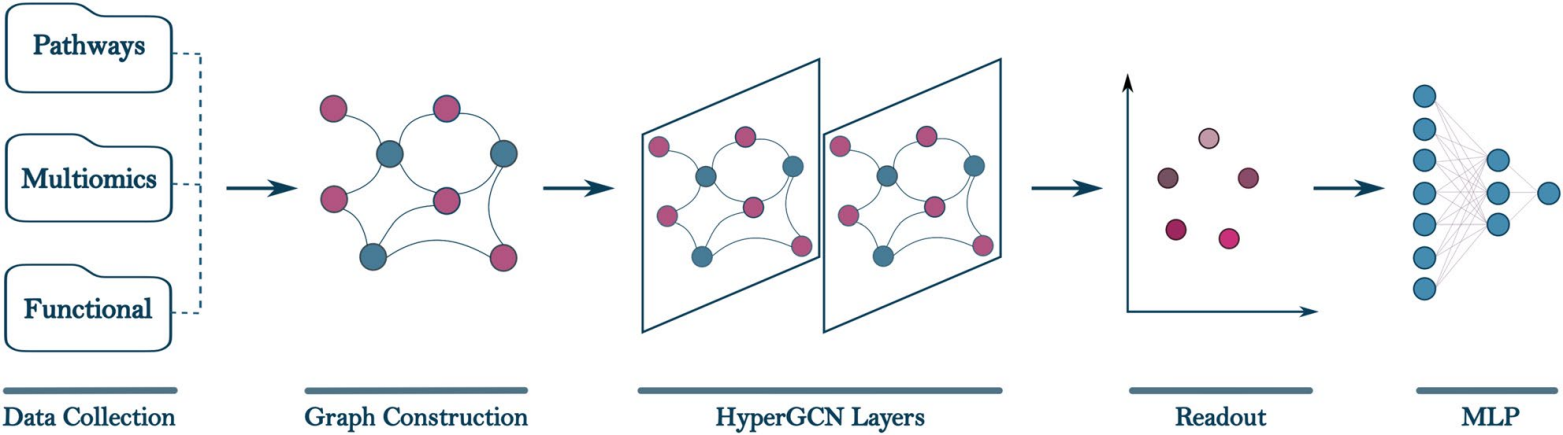
Overview of the ONCOPLEX framework, illustrating the end-to-end workflow from data integration and pathway-based hypergraph construction to representation learning and cancer driver gene prediction.

ONCOPLEX is a pathway-informed hypergraph-based framework designed to prioritize candidate cancer driver genes by integrating multi-omics features with higher-order biological relationships among genes. An overview of the ONCOPLEX pipeline is presented in Fig. 1, which illustrates the key components, ranging from data integration and hypergraph construction to representation learning and final prediction. Detailed descriptions of the model architecture, data preprocessing, and evaluation protocols are provided in the Methods section. In this section, we evaluate the performance of ONCOPLEX in both pan-cancer and cancer-type-specific settings using datasets derived from The Cancer Genome Atlas (TCGA). Model performance is assessed using standard classification and ranking metrics, including the area under the receiver operating characteristic curve (AUROC), the area under the precision–recall curve (AUPRC), and the F1-score. Comparisons are conducted against several state-of-the-art graph and hypergraph-based methods for the prediction of cancer driver genes, including EMOGI, MTGCN, HGCN, and DISHyper.

### ONCOPLEX shows superior performance over competing methods on pan-cancer data

**Fig. 2:**
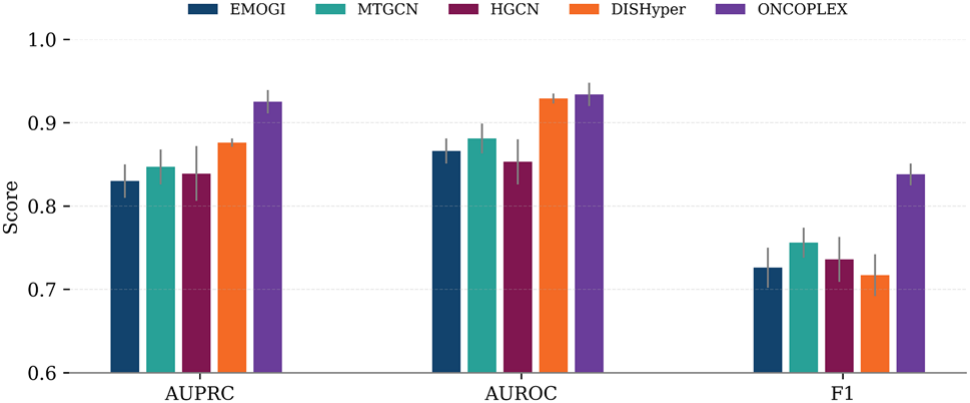
Comparison of five methods, EMOGI, MTGCN, HGCN, DISHyper, and ONCOPLEX, on pan-cancer driver gene prediction using three performance metrics: AUPRC, AUROC, and F1-score. Error bars indicate standard deviations across evaluation runs. ONCOPLEX consistently achieves the highest scores across all metrics.

We compared ONCOPLEX with four representative baseline methods (EMOGI, MTGCN, HGCN, and DISHyper) in the pan-cancer driver gene prediction task using three evaluation metrics: AUPRC, AUROC, and F1-score. As shown in Fig. 2, ONCOPLEX consistently outperforms all competing methods across all metrics. The detailed numerical performance values corresponding to this comparison are reported in Supplementary Table S1. It achieved the highest AUPRC of $0.925\,\pm \,0.014$, notably outperforming DISHyper, the second-best method ($0.876\,\pm \,0.005$). ONCOPLEX also obtained the best AUROC ($0.934\,\pm \,0.014$) and F1-score ($0.838\,\pm \,0.013$), indicating superior precision and recall in identifying cancer driver genes. These results underscore the effectiveness of our integrated hypergraph framework in modeling complex relationships among multi-omics features across diverse cancer types.

### ONCOPLEX maintains superior performance across individual cancer types

**Fig. 3:**
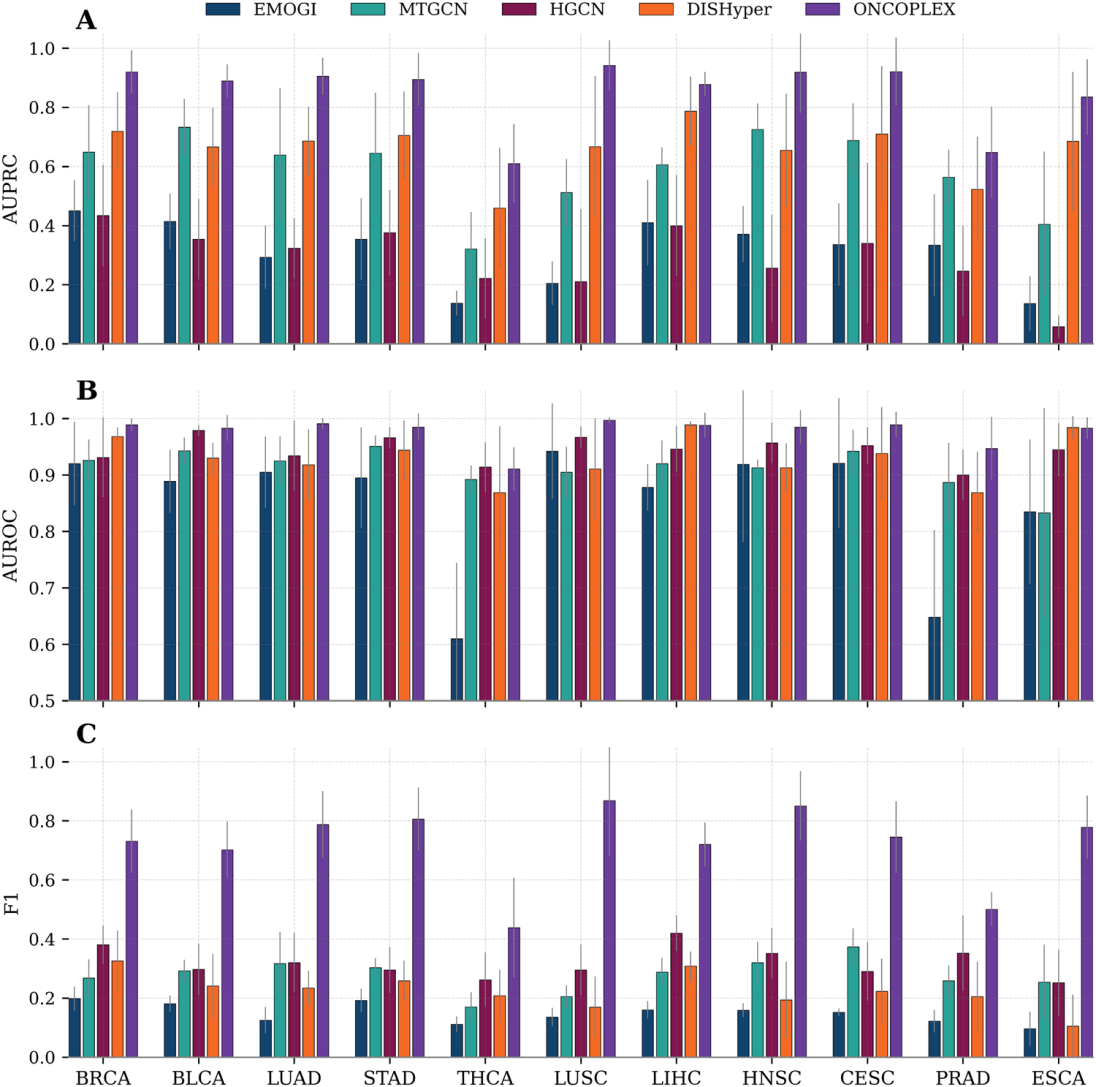
Performance comparison of five methods (EMOGI, MTGCN, HGCN, DISHyper, and ONCOPLEX) for cancer driver gene prediction across 11 individual cancer types. **A** AUPRC, **B** AUROC, and **C** F1-score for each method. Bars denote mean values across multiple evaluation runs, with error bars representing standard deviations. Across all three evaluation metrics, ONCOPLEX shows superior performance on most cancer types, indicating strong robustness and generalizability.

To assess the generalizability of ONCOPLEX in different tumor contexts, we evaluated its performance in 11 individual cancer types: breast invasive carcinoma (BRCA), bladder urothelial carcinoma (BLCA), lung adenocarcinoma (LUAD), stomach adenocarcinoma (STAD), thyroid carcinoma (THCA), lung squamous cell carcinoma (LUSC), liver hepatocellular carcinoma (LIHC), head and neck squamous cell carcinoma (HNSC), cervical squamous cell carcinoma and endocervical adenocarcinoma (CESC), prostate adenocarcinoma (PRAD), and esophageal carcinoma (ESCA). These cancer types were selected because they have at least 15 curated driver genes, ensuring sufficient labeled data for reliable training and evaluation, and because they are commonly used in prior studies on cancer driver gene prediction, enabling fair benchmarking and direct comparison with existing methods. As illustrated in Fig. 3**(A–C)**, ONCOPLEX exhibits superior performance relative to the four baseline methods (EMOGI, MTGCN, HGCN, and DISHyper) in the three evaluation metrics: AUPRC, AUROC, and F1-score. Corresponding numerical results for each cancer type are provided in Supplementary Table S2. In particular, ONCOPLEX achieved the highest AUPRC in all 11 cancer types, indicating its superior ability to distinguish true driver genes from nondrivers under class imbalance. Specifically, it attained an AUPRC of 0.942 in LUSC and 0.921 in CESC, significantly outperforming the second-best methods by wide margins. Similarly, ONCOPLEX obtained the best AUROC scores in most cancer types, with values exceeding 0.98 in LUAD, LUSC, and LIHC. This highlights its strong discriminative power to classify positive and negative examples. In terms of the F1-measure, which balances precision and recall, ONCOPLEX again achieved the highest values in all types of cancer. Its performance was particularly notable in LUAD (0.787), LUSC (0.868), and HNSC (0.850), reflecting its effectiveness in correctly identifying driver genes without sacrificing precision.

These results demonstrate that ONCOPLEX is not only effective in the pan-cancer setting but also highly robust when applied to individual cancer types with varying mutational landscapes and omics profiles. This emphasizes the advantage of integrating pathway-informed hypergraph topology with rich and heterogeneous biological features in a unified framework.

### ONCOPLEX ranks cancer driver genes with high precision

**Fig. 4:**
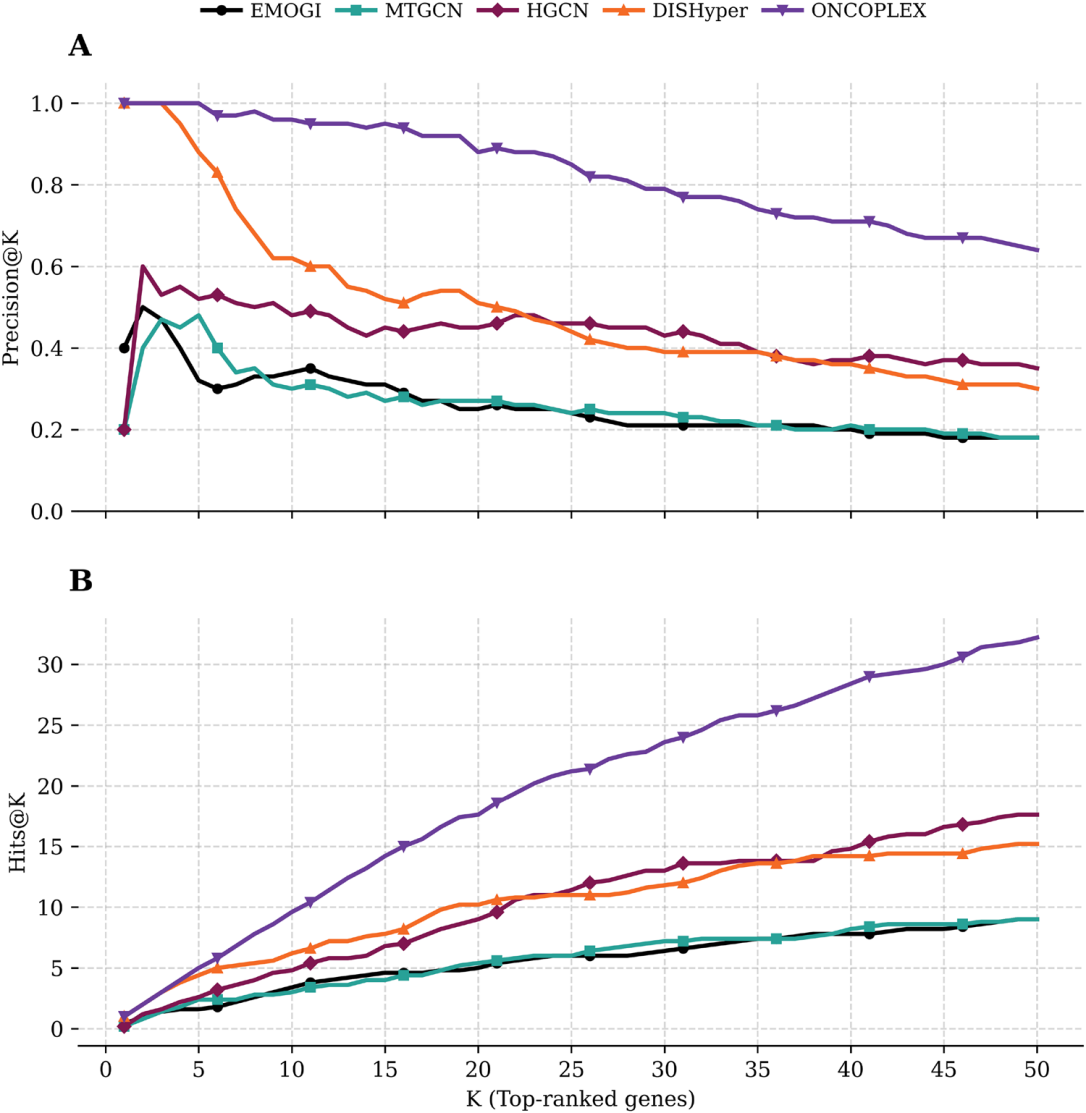
Performance comparison of ONCOPLEX and baseline methods for pan-cancer driver gene ranking. **A** Precision@K and **B** Hits@K for identifying known cancer driver genes among the top-K ranked genes in a unified pan-cancer setting. Five methods (EMOGI, MTGCN, HGCN, DISHyper, and ONCOPLEX) are evaluated for K ranging from 1 to 50.

We benchmarked ONCOPLEX against four representative methods (EMOGI, MTGCN, HGCN, and DISHyper) using Precision@K and Hits@K metrics in the pan-cancer setting, with K ranging from 1 to 50, as shown in Fig. 4 (**A** for Precision@K and **B** for Hits@K). Numerical values can be found in Supplementary Table S3. ONCOPLEX maintains superior performance across the full range of K, and the performance gap is especially pronounced at smaller values of K, where the correct identification of the candidates for the most confident driver gene is the most critical. Among the baselines, DISHyper tracks the closest to ONCOPLEX, highlighting the strength of hypergraph-based modeling. However, ONCOPLEX retains a clear and consistent lead in all values of K, thanks to its integration of diverse omics features and its effective hypergraph convolutional architecture. HGCN, another hypergraph model, performs moderately, but lags behind both ONCOPLEX and DISHyper, likely due to its simple architecture and the inability to effectively exploit the rich structure of the hypergraph. In contrast, the graph-based models MTGCN and EMOGI show weaker overall performance, reaffirming that hypergraph-based approaches better capture complex higher-order gene interactions. These findings support ONCOPLEX as a powerful method for classifying cancer driver genes on a global scale, encompassing all cancers (pan-cancer).

**Fig. 5:**
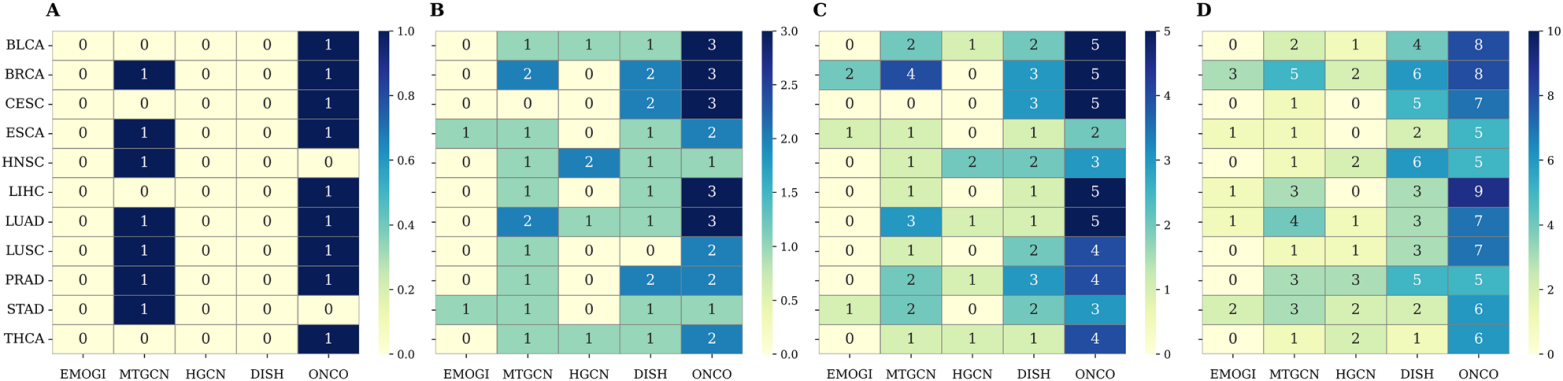
Cancer-specific driver gene ranking performance evaluated using Hits@K across 11 cancer types. Panels **A**–**D** show heatmaps of Hits@1, Hits@3, Hits@5, and Hits@10, respectively. Each cell indicates the number of known cancer driver genes recovered among the top-K ranked genes for a given method and cancer type, highlighting differences in ranking effectiveness across methods and cancers.

To evaluate driver gene prioritization in cancer-type-specific contexts, we further assessed Hits@K performance for K = 1, 3, 5, and 10 in 11 types of cancer (Fig. 5; **A–D** correspond to Hits@1, Hits@3, Hits@5, and Hits@10, respectively). This complements the evaluation of pan-cancers and shows how well each method performs in more focused clinical settings. At Hits@1, ONCOPLEX correctly ranks a known driver gene first in 9 out of 11 cancer types, considerably higher than the best-performing baseline MTGCN, which achieves this in 7 cancers, while DISHyper, HGCN, and EMOGI record zero Hits@1. At Hits@3, ONCOPLEX reaches the maximum score of 3 in 5 cancer types, showing strong top-rank precision. DISHyper achieves 2 correct hits in 3 types, MTGCN in 2 types, HGCN in 1 cancer type, and EMOGI never exceeds 1.

Looking beyond the very top ranks, ONCOPLEX maintains its superiority at Hits@5 and Hits@10. It consistently ranks the 4 to 5 known driver genes in the top 5 positions for many cancers, while other methods typically fall below 3. At Hits@10, ONCOPLEX often reaches 7–9 correct genes, compared to the best case of 5-6 from MTGCN or DISHyper. This illustrates that ONCOPLEX not only excels in identifying the most critical driver genes, but also maintains precision deeper into the ranked list. Although MTGCN performs relatively well in Hits@1 in this setting, better than it did in pan-cancer, it still lags behind ONCOPLEX at higher K values. Meanwhile, DISHyper shows competitive performance in the midrange K, but never surpasses ONCOPLEX in any cancer. These results collectively reinforce ONCOPLEX’s robust ability to prioritize cancer driver genes in both pan-cancer and cancer-type-specific tasks.

### ONCOPLEX benefits from effective integration of multi-source node features

**Fig. 6:**
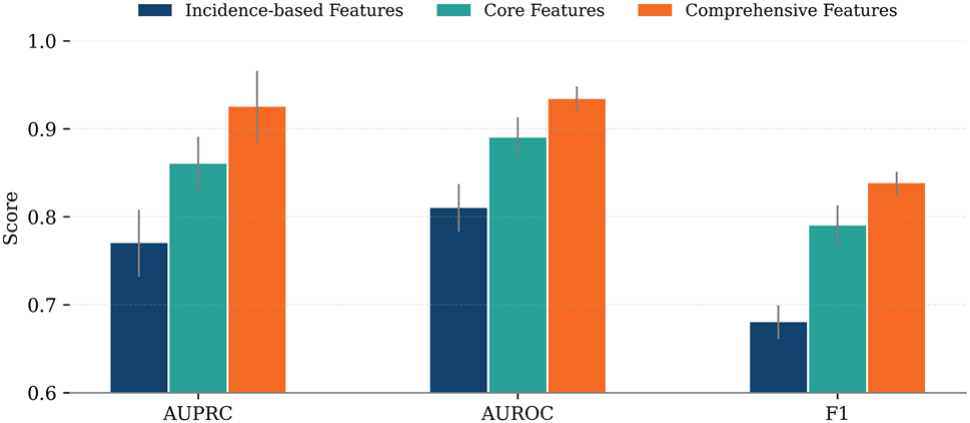
Ablation study on pan-cancer data examining the contribution of different input feature sets to model performance. Results are reported across multiple evaluation metrics; bars indicate mean scores over cross-validation folds, with error bars representing the corresponding standard deviations.

**Fig. 7:**
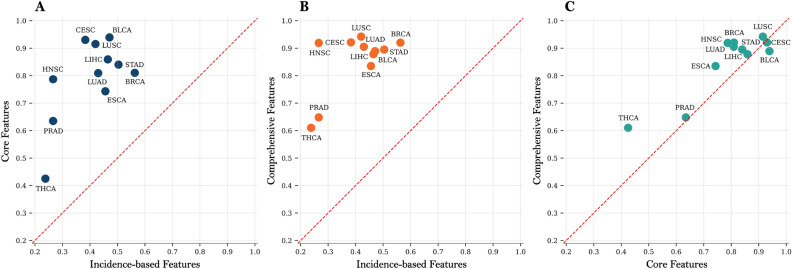
Cancer-type-specific ablation analysis comparing model performance (AUPRC) across different node feature sets. Each point corresponds to a cancer type. Panels **A**–**C** show pairwise comparisons of Incidence-based features versus Core omics features, Incidence-based features versus Comprehensive features, and Core omics features versus Comprehensive features, respectively. The red dashed line indicates the identity line ($y=x$).

To assess the contribution of different types of node features, we performed an ablation study in two complementary settings: pan-cancer and cancer-type-specific prediction tasks. The results are shown in Figs. 6 and 7, respectively.

**Table 1:** Top 3 predicted driver genes per cancer type, as ranked by ONCOPLEX after filtering out well-established cancer-specific drivers.

Cancer	Gene	Rank	Common Driver?	Database Support
BRCA	GRB2	3	No	CancerMine
	MAPK1	5	Yes	CancerMine, OncoKB
	MAPK3	6	No	CancerMine
BLCA	AKT1	2	Yes	Ongene, CancerMine, OncoKB
	PIK3R1	3	Yes	Ongene, CancerMine, OncoKB
	MAPK1	4	Yes	CancerMine, OncoKB
LUAD	AKT1	2	Yes	Ongene, CancerMine, OncoKB
	SOS1	6	No	CancerMine
	GRB2	11	No	CancerMine
STAD	AKT1	2	Yes	Ongene, CancerMine, OncoKB
	HRAS	3	Yes	Ongene, CancerMine, OncoKB
	SHC1	17	No	CancerMine
THCA	NPM1	2	Yes	Ongene, CancerMine, OncoKB
	MAP2K1	3	Yes	CancerMine, OncoKB
	MAPK3	4	No	CancerMine
LUSC	AKT2	4	Yes	Ongene, CancerMine, OncoKB
	SOS1	8	No	CancerMine
	RAF1	9	Yes	Ongene, CancerMine, OncoKB
LIHC	AKT1	2	Yes	CancerMine, OncoKB
	HRAS	3	Yes	Ongene, CancerMine, OncoKB
	SMAD4	4	Yes	CancerMine, OncoKB
HNSC	KRAS	2	Yes	Ongene, CancerMine, OncoKB
	AKT1	3	Yes	Ongene, CancerMine, OncoKB
	BRAF	4	Yes	Ongene, CancerMine, OncoKB
CESC	MAPK3	2	No	CancerMine
	HRAS	3	Yes	Ongene, CancerMine, OncoKB
	MAP2K1	4	Yes	CancerMine, OncoKB
PRAD	MAPK1	2	Yes	CancerMine, OncoKB
	MAP2K1	4	Yes	CancerMine, OncoKB
	GRB2	5	No	CancerMine
ESCA	PIK3R1	2	Yes	Ongene, CancerMine, OncoKB
	CCND1	7	Yes	Ongene, CancerMine, OncoKB
	GSK3B	10	No	CancerMine, OncoKB

In the context of pan-cancer (Fig. 6; see Supplementary Table S4 for detailed results), we evaluated the performance of the model using three metrics, the AUPRC, the AUROC, and the F1 score, in three configurations of characteristics: incidence-based, core, and comprehensive features. Performance improved consistently across all metrics as we moved from incidence-based to core features and further to comprehensive features. For example, AUPRC increased from 0.77 (±0.038) with incidence-based characteristics to 0.86 (±0.031) with core characteristics and reached 0.925 (±0.041) with comprehensive characteristics. Similarly, AUROC increased from 0.81 to 0.89 and then to 0.934, while the F1-score improved from 0.68 to 0.79 and finally to 0.838. These improvements highlight the cumulative benefits of integrating increasingly informative biological features.

This trend is further confirmed in the cancer type-specific analysis (Fig. 7A–C, comparing incidence-based vs core features, incidence-based vs comprehensive features, and core vs comprehensive features, respectively; numerical values in Supplementary Table S5). Points above the identity line indicate cases where the feature set on the vertical axis outperforms the one on the horizontal axis. In most types of cancer, the core and comprehensive features outperformed the incidence-based features (left and middle panels), and the comprehensive features also consistently outperformed the core features (right panel). In particular, using comprehensive features achieved a higher AUPRC in 10 of 11 cancer types compared to core features.

Together, these results underscore the importance of effective feature integration, moving beyond topological signals to incorporate molecular, genomic, and phenotypic data to improve cancer driver gene prediction in both pan-cancer and cancer-specific settings.

**Fig. 8:**
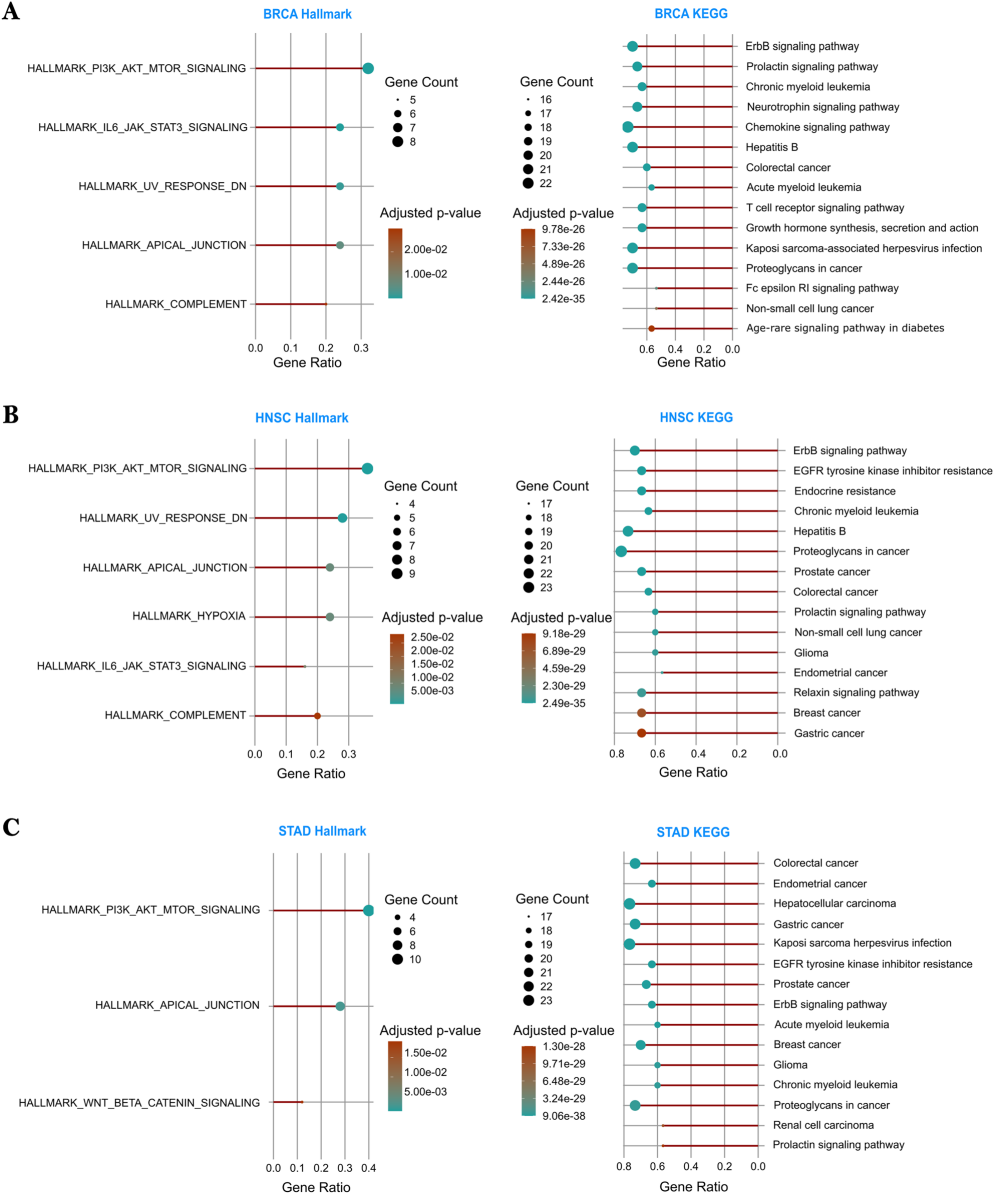
Functional enrichment analysis of top-ranked genes predicted by ONCOPLEX for three cancer types. Panels **A**–**C** correspond to BRCA, HNSC, and STAD, respectively. For each cancer type, significantly enriched KEGG pathways (right) and Hallmark gene sets (left) are shown based on adjusted $p < 0.05$.

### Discovery of cancer driver genes and enrichment analysis

ONCOPLEX discovered key driver genes that contribute to cancer development, including general and cancer-type-specific driver genes. Table 1 presents the three main predicted candidate genes for each type of cancer after excluding previously known and well-characterized driver genes (the complete gene list is provided in Supplementary Table S6).

In BRCA, GRB2, MAPK1, and MAPK3 were identified as strong candidates based on their high rankings. Although MAPK1 is a well-known pan-cancer driver, it has not been specifically reported as a BRCA driver. However, it appears in cancer-related databases such as CancerMine. GRB2 and MAPK3, although not established BRCA drivers, have supportive evidence in the literature. GRB2 has been shown to protect stagnant replication forks, maintaining genome stability, and is considered a potential biomarker^21^. MAPK3, a member of the MAPK gene family, regulates various cellular processes, and its variants can disrupt cancer-relevant signaling pathways^22^. In BLCA, all three predicted genes were observed: MAPK1, AKT1, and PIK3R1, which are established drivers according to multiple databases. In particular, PIK3R1 is negatively regulated in tumor tissues compared to normal tissues and functions within the PI3K / AKT signaling pathway along with MAPK1 and SRC. It has been explored as a target for chemotherapy and as a diagnostic biomarker^23,24^. In HNSC, ONCOPLEX identified well-established drivers: KRAS, AKT1, and BRAF. KRAS promotes HNSC progression by increasing Runx1, which enhances oral epithelial proliferation and migration while inhibiting apoptosis^25^. BRAF, mutated in more than 60% cancers, is often overexpressed in HNSC, COAD, and LUSC^26^.

Across cancer types, ONCOPLEX tended to highly rank known pancancer genes, even if not previously reported as drivers of a specific cancer type, due to shared molecular pathways. However, the model also highlighted potential candidates. For example, GRB2 and MAPK3 in BRCA, SHC1 in STAD, and GSK3B in ESCA were not labeled as canonical drivers, but they have supportive cancer associations in CancerMine and were ranked among the top candidates. Furthermore, ONCOPLEX identified genes such as GAB1 in BRCA and UBA52 in BLCA that have not yet been included in known cancer gene databases and are supported by indirect functional evidence (see Supplementary Table S7). We consider these as entirely novel candidate drivers.

To understand the biological roles of the predicted genes, we performed a Hallmark and pathway enrichment analysis on the 30 highest-ranked genes using the clusterProfiler R package^27^. Figure 8 (A–C for BRCA, HNSC, and STAD, respectively) illustrates the terms most significantly enriched ($\text {adjusted } p$-value $< 0.05$) for BRCA, HNSC, and STAD. In particular, in BRCA, predicted genes were enriched in the ErbB signaling pathway, followed by the prolactin signaling pathway and the chronic myeloid leukemia pathway. These pathways also exhibited high gene counts among the top 30 genes and are strongly associated with breast cancer. Hallmark analysis highlighted pathways such as PI3K_AKT_MTOR and JAK_STAT3 signaling. In HNSC, the top enriched pathways included ErbB signaling, resistance to EGFR tyrosine kinase inhibitors, and the prostate cancer pathway. These genes were also enriched in Hallmark sets shared with BRCA, except for the addition of the unique HYPOXIA signature of HNSC. In STAD, enriched pathways included the colorectal and breast cancer pathways, as well as the WNT signaling pathway, which was enriched for STAD and not prominent in BRCA or HNSC. Hence, STAD showed fewer enriched Hallmark terms compared to the other two.

In particular, the ErbB signaling pathway was consistently enriched in all three types of cancer, emphasizing its central role in tumor progression and serving as a potential target for cancer treatment^28^. In addition to cancer-related pathways, immune-associated pathways such as T cell receptor signaling were also significantly enriched. This may reflect the immune-active tumor microenvironment and suggests the potential for immunotherapy strategies, especially in BRCA and HNSC. Additional analyses and extended results are provided in Supplementary Tables S8–S11.

## Discussion

In recent years, the prediction of cancer driver mutations has remained a significant challenge in cancer genomics, despite the development of numerous computational methods. This difficulty arises from the heterogeneity of the disease and the complexity of its underlying molecular mechanisms. In the previous sections, we presented the key findings of our study that aimed to tackle this issue through a hypergraph-based approach.

First, we evaluated our hypergraph model on a pan-cancer dataset, achieving superior performance in terms of AUPRC, AUROC, F1, and ranking metrics compared to both graph-based and hypergraph-based benchmark methods. The overall performance of our model was more reliable in gene ranking tasks, demonstrating the effectiveness of higher-order representations in recovering known cancer genes, especially compared to graph-based models such as EMOGI and MTGCN.

Given the diversity of driver genes across specific cancer types, we further investigated the performance of our model in individual cancer datasets. Despite the challenges posed by the limited number of known driver genes in certain cancers, our model consistently showed strong and reliable performance. As shown in Fig.  3, the best results were obtained for BRCA, LUSC, and CESC, probably due to their larger number of known driver genes or shared pathways. In particular, BRCA has been extensively studied, and its driver mutations are better characterized than those of other cancers. Furthermore, we found that in cancer types with fewer known driver genes, such as ESCA, which has the lowest number of known driver genes compared to other cancers (18 known driver genes in the training set), ONCOPLEX still achieved high performance (AUPRC of 0.83). This can be attributed to the involvement of ESCA in more than 60 shared pathways with other cancers, enabling the model to extract meaningful signals from related contexts, even with limited direct supervision.

Considering that the primary goal of disease gene prioritization is the identification of new potential genes, our model successfully prioritized several candidate cancer genes, as listed in Table 1, many of which have been previously implicated in cancer studies. These genes are directly or indirectly involved in cancer-related pathways. For instance, MAPK1, MAPK3, and well-established oncogenes such as KRAS and BRAF have been linked to cancer development through the activation or suppression of critical signaling pathways. In particular, we identified several highly ranked predicted genes that had not previously been reported in established cancer gene databases. For example, members of the ADCY gene family were predicted in LIHC. A search of the PubMed literature revealed that these genes are involved in various diseases due to their role in the regulation of key cellular functions. Among these, some ADCY genes have been associated with liver-related diseases, suggesting a possible link to liver tumor progression^29^. This finding is promising, as it highlights the model’s ability to not only identify genes directly linked to the tumor but also uncover indirectly associated genes that may contribute to tumor development through broader biological mechanisms.

In addition to identifying new candidates, validation of their biological and clinical relevance is essential, either experimentally or through bioinformatics tools. Our analysis showed that the predicted genes for 11 types of cancer were significantly enriched in cancer-related pathways and ontology, including hallmark pathways such as Pathways in Cancer, immune-related pathways, and pathways regulating essential cell functions. These findings suggest that our predicted genes are functionally relevant and share biological mechanisms with known drivers. This further supports the role of pathway-level information in the construction of biologically significant cancer gene networks. In addition, this highlights the value of higher-order relationships in the prioritization of disease genes.

However, in our cancer-specific analysis, we observed a limitation related to the extensive overlap of the pathways. Cancers such as HNSC are involved in more than 500 pathways, the highest among all types of cancer in our data set. This abundance of shared pathways made it difficult for the model to capture cancer-type-specific drivers. Instead, the model tends to prioritize well-known, commonly pan-cancer genes such as KRAS, NRAS, and JUN, which appear in many cancers, while failing to recover top-ranked, type-specific driver genes. This phenomenon suggests that cancer-specific mutations and biological mechanisms may be highly similar, making a pan-cancer study sufficient to capture common cancer drivers in multiple cancer types, as previously observed in^30^. As a result, the reliance of our model on shared pathways may inadvertently obscure cancer-type-specific signals, which aligns with our observations in several cancer types. However, while shared pathways provide valuable context for learning, they can also dilute signals that are unique to individual cancer types. This underscores the need for more customized pathway integration strategies in future work.

Beyond predictive accuracy, the clinical relevance of ONCOPLEX predictions presents significant opportunities for translational research. The high-ranking candidate genes identified by our model, many of which are supported by pathway enrichment and evidence from the literature, can inform the discovery of targets in drug development, especially for therapies aimed at key signaling nodes or synthetic lethal partners of known drivers. Furthermore, ONCOPLEX’s ability to prioritize genes based on multiomics and pathway context positions it as a valuable tool for identifying biomarkers in precision cancer, enabling more personalized treatment strategies. Moreover, the strong performance of the model in cancer types with limited known drivers demonstrates its potential application in rare or understudied cancers, where traditional data-driven methods struggle due to sparse supervision. This highlights ONCOPLEX’s utility not only in hypothesis generation but also in guiding downstream experimental validation and clinical decision making.

## Conclusion

In this study, we presented ONCOPLEX, a hypergraph-based deep learning framework designed to prioritize cancer driver genes by modeling complex, higher-order gene interactions informed by biological pathways. By integrating diverse multi-omics features within a unified hypergraph structure, ONCOPLEX captures both local and global biological contexts, enabling more accurate and interpretable driver gene prediction.

Through comprehensive evaluation across pan-cancer and cancer-type-specific datasets, ONCOPLEX demonstrated consistent superiority over existing graph- and hypergraph-based baseline methods. It achieved strong predictive accuracy, particularly in cancers with limited known drivers, and exhibited robust generalization across cancer types. Importantly, the top-ranked gene predictions were significantly enriched in hallmark cancer pathways and supported by evidence from the literature, highlighting their biological relevance and translational potential.

Despite these promising results, we observed challenges in distinguishing cancer-type-specific driver genes in cancers with extensive pathway overlap, where shared signaling mechanisms may obscure unique mutational signals. Addressing this limitation requires more refined strategies to integrate pathway knowledge in a context-specific way.

In the future, ONCOPLEX will provide a solid foundation for next-generation tools in cancer genomics. Our work will focus on refining hypergraph construction with patient-specific data, incorporating dynamic pathway activity, and extending the model to longitudinal and single-cell applications. Ultimately, ONCOPLEX represents a step toward more precise, biologically grounded computational frameworks aligned with the goals of personalized oncology and targeted therapeutic discovery.

## Method

To address the limitations of existing methods in cancer driver gene prediction, ONCOPLEX is proposed. This unified framework integrates diverse genomic and functional data using a hypergraph-based graph neural network. Before detailing the model architecture, the hypergraph structure is first defined, and the cancer driver prediction problem is formalized.

### Problem formulation and preliminaries

A hypergraph is a generalization of a graph in which the edges, called hyperedges, can connect any number of nodes. This characteristic makes hypergraphs particularly well-suited for modeling higher-order gene relationships, such as co-participation in biological pathways.

Formally, let $G = (V, E, W, X)$ denote a weighted hypergraph, where $V = \{v_1, v_2, \ldots , v_n\}$ is the set of nodes, each representing a gene; $E = \{e_1, e_2, \ldots , e_m\} \subseteq 2^V$ is the set of hyperedges, each corresponding to a biological pathway involving multiple genes; $W \in \mathbb {R}^{m \times m}$ is a diagonal matrix where $W_{ii}$ denotes the weight of the hyperedge $e_i$; and $X \in \mathbb {R}^{n \times d}$ is the node feature matrix, with $X_i$ denoting the feature vector of node $v_i$. The structure of the hypergraph is encoded by an incidence matrix $H \in \mathbb {R}^{n \times m}$, where $H(i, j) = 1$ if the node $v_i$ is a member of the hyperedge $e_j$, and 0 otherwise. Based on this, the degree matrix of the node $D_v \in \mathbb {R}^{n \times n}$ is defined with entries $D_v(i, i) = \sum _{j=1}^m H(i, j)$, and the hyperedge degree matrix $B \in \mathbb {R}^{m \times m}$ is defined with entries $B(j, j) = \sum _{i=1}^n H(i, j)$.

The hypergraph convolutional neural network (HyperGCN^31^) extends the message-passing frameworks by allowing each node to aggregate information from other nodes within the same hyperedges. At each layer $l$, the node representations are updated according to:

$$X^{(l+1)} = \sigma \left( D_v^{-1/2} H W B^{-1} H^\top D_v^{-1/2} X^{(l)} \Theta \right) ,$$

where $X^{(l)} \in \mathbb {R}^{n \times d}$ is the input feature matrix in layer $l$, $\Theta \in \mathbb {R}^{d \times d'}$ is a trainable weight matrix, and $\sigma (\cdot )$ is a non-linear activation function such as ReLU.

Given the attributed hypergraph $G = (V, E, W, X)$ and binary labels $Y \in \{0, 1\}^m$ for a subset of genes $\{v_1, \ldots , v_m\}$, the objective is to learn a function $M: V \rightarrow [0,1]$ that predicts the probability that each gene is a cancer driver. The model should effectively leverage both the topological structure of the hypergraph and the node features to prioritize candidate driver genes for further investigation.

### Overview of the ONCOPLEX framework

Figure 1 illustrates the ONCOPLEX pipeline, which consists of five main components:

- **Data collection and processing**: Multiomics profiles such as somatic mutations, gene expression, and DNA methylation, along with functional annotations, are obtained from public cancer datasets. The features are normalized and aligned in the samples.
- **Graph construction**: Each node represents a gene, and each hyperedge encodes a higher-order relationship among multiple genes that participate in the same biological pathway.
- **Graph neural network**: A hypergraph convolutional network (HyperGCN) propagates feature information through the high-order structure to learn expressive node representations.
- **Readout**: For each node, embeddings from different layers are aggregated to form a unified representation. The readout function also ensures that all node embeddings have the same dimensionality.
- **MLP for prediction**: A multilayer perceptron (MLP) estimates the probability that each gene is a cancer driver based on its learned representation.

### Data collection and preprocessing

Three main types of data were collected: pathway data to define the graph structure, molecular and biological features to initialize node attributes, and curated gene labels for supervised learning in cancer driver gene prediction.

**Pathway data for graph topology construction:** A total of 3,656 biological pathways were curated from the KEGG, Reactome, BioCarta, and PID databases^32^, excluding cancer-specific terms (e.g., cancer, tumor) to reduce potential bias. Each pathway was treated as a hyperedge connecting the genes involved in the underlying biological process. These pathways form the hypergraph topology, where genes are nodes and pathways are hyperedges.

**Molecular and biological data for node features:** Each gene was initialized with a feature vector derived from three sources:

- **Incidence-based features:** Binary vectors encoding the membership of each gene in all pathways, capturing topological information from the hypergraph structure without any biological annotation.
- **Core omics features:** Somatic mutations, gene expression, and DNA methylation profiles of 16 cancer types were collected from TCGA^33^. Specifically, mutation features were computed as non-silent mutation frequency normalized by gene length (excluding ultra-mutated samples). Expression features were calculated as log$_2$ fold changes between tumor and normal tissues matched using normalized FPKM values, and methylation features were derived as differences in beta values between tumor and normal samples. For pan-cancer graphs, features from the three omics layers in 16 cancer types were concatenated to form a 48-dimensional vector per gene. For cancer-specific graphs, only the three omics features relevant to the specified cancer type were used (see Supplementary Material, Subsection 1.2).
- **Comprehensive biological features:** A set of 44 features that include variant pathogenicity score, evolutionary conservation, histone modifications, and other functional descriptors curated from previous studies^34^. These features help to distinguish oncogenes from tumor suppressor genes (see Supplementary Table S12 for the full list).

**Cancer driver gene labels:** Driver gene annotations were obtained from two well-established resources: the Cancer Gene Census (CGC) and the Network of Cancer Genes (NCG). For each type of cancer, genes labeled as drivers in either database were treated as positive instances, while all remaining genes were considered negative. These labels were used to train and evaluate ONCOPLEX in both pan-cancer and cancer-type-specific settings (see the Supplementary Table S13 for detailed counts).

In the context of pan-cancer, a set of positive genes was compiled by integrating multiple curated sources. Specifically, 711 genes were included from the NCG database, which provides manually curated lists of known and candidate cancer driver genes. In addition, cancer-related genes identified by DigSEE, a database that extracts gene-cancer associations through PubMed abstract literature mining, were incorporated. Defining the negative class is more challenging, as many genes remain poorly characterized and could potentially be associated with cancer. To construct a reliable negative set, a conservative three-step exclusion strategy was applied following^14^: (1) all genes labeled as cancer drivers were excluded, (2) genes participating in cancer-related KEGG pathways were removed, and (3) genes predicted to be cancer-associated by previous computational models were filtered out. After this filtering, 753 positive and 1,116 negative genes present in the constructed hypergraph were retained. The remaining genes were treated as unlabeled, allowing ONCOPLEX to potentially identify novel candidate driver genes. This conservative strategy reduces label noise by minimizing the risk of incorrectly assigning potential cancer-associated genes to the negative class and is consistent with the negative-set construction used in prior benchmarking studies, enabling fair and meaningful comparisons with existing methods.

For cancer-type-specific experiments, a similar approach was followed. Driver genes were collected for each type of cancer from COSMIC and DigSEE. To ensure sufficient labeled data for learning, cancer types with fewer than 15 driver genes were excluded, resulting in 11 cancer types included in the analysis. The negative class remained consistent with the pan-cancer setting. In particular, the number of known driver genes for individual cancer types is considerably smaller than the number of presumed passenger genes, further motivating the need for models capable of discovering novel drivers from sparse supervision.

### Graph construction

To model high-order gene relationships, a hypergraph was constructed using curated pathways from the KEGG, Reactome, BioCarta, and PID databases, excluding cancer-related terms to avoid bias. The final hypergraph serves as the structural backbone for ONCOPLEX, comprising the following components:

- **Graph topology:** Each node represents a gene and each hyperedge corresponds to a biological pathway connecting multiple genes. In total, the hypergraph contains 13,560 nodes and 3,656 hyperedges, capturing a wide range of biological processes.
- **Node features:** Each node is initialized with biologically informed characteristics derived from three categories: (1) incidence-based characteristics that capture structural functions in the hypergraph, (2) core omics characteristics, including TCGA mutation, expression, and methylation data, and (3) comprehensive features representing genomic and functional annotations.
- **Edge weights:** To incorporate cancer relevance, each hyperedge is assigned a weight based on the number of known cancer driver genes it contains for a specific cancer type. For example, pathways with multiple known breast cancer drivers are given higher weights when constructing the hypergraph for breast cancer. It is important to note that in the cross-validation setting, hyperedge weights are computed independently within each fold using only driver gene labels from the training set. Driver genes assigned to validation or test sets are not used during hyperedge construction or weighting. These weights guide the model toward biologically meaningful patterns during training.

The resulting weighted hypergraph is then used as input to the ONCOPLEX model for both pan-cancer and cancer-type-specific prediction tasks.

### Model architecture

ONCOPLEX is built on a hypergraph neural network framework designed to capture complex, high-order interactions among genes. The architecture comprises the following key components:

- **Hypergraph convolution:** HyperGCN is adopted to propagate information across the hypergraph structure, enabling each gene to aggregate signals from other genes that co-occur in shared biological pathways. This allows the model to learn informative node embeddings that integrate both multi-omics features and pathway context.
- **Readout function:** To incorporate global structural information, a readout function is applied that aggregates the intermediate embeddings from multiple HyperGCN layers and forms a unified fixed-dimensional representation for each gene. This step ensures consistent embedding dimensionality across the network and supports robust downstream prediction.
- **Prediction module:** The representations of the resulting nodes are passed through a multilayer perceptron (MLP), which outputs the probability that each gene is a cancer driver. The model is trained using binary cross-entropy loss based on known driver and non-driver gene labels.

### Training and evaluation protocols

ONCOPLEX was trained using binary cross-entropy loss to distinguish driver genes from passengers. The model was optimized with the AdamW optimizer^35^, and early stopping was applied based on validation loss to prevent overfitting. Five-fold cross-validation was conducted for both pan-cancer and cancer-specific experiments, ensuring class balance in each fold. In each round, one fold was used as the test set, and the remaining four were used for training and validation. Specifically, an inner four-fold cross-validation was employed within the training folds to select optimal hyperparameters. All experiments were repeated with five random seeds to ensure robustness, and the mean and standard deviation of the AUROC, AUPRC, and F1 score across all test folds and seeds are reported. Hyperparameters such as learning rate, dropout rate, and hidden layer dimensions were tuned via grid search using the inner validation sets (for more details, see Supplementary Subsection 1.3).

### Baselines for comparison

ONCOPLEX was compared with four representative graph-based methods for cancer driver gene prediction. All models were retrained using our curated feature sets and evaluated under the same experimental protocol to ensure fairness.

- **EMOGI**^14^: A graph neural network that integrates multi-omics data (mutations, expression, and methylation) using a fixed protein–protein interaction (PPI) network. In our setup, EMOGI was retrained with our core omics features and the STRING PPI network.
- **MTGCN**^15^: A multitask GCN framework that captures cancer-type-specific patterns by jointly learning across multiple cancer types. MTGCN was retrained using the core omics features and a PPI-derived gene graph.
- **HGCN**^36^: a convolutional hypergraph model originally proposed for hypergraph learning. The HGCN architecture was implemented and applied to the pathway-based hypergraph using the same node features as ONCOPLEX. This baseline isolates the effect of model architecture using our graph construction.
- **DISHyper**^20^: A hypergraph-based method that constructs weighted, cancer-specific hyperedges based on gene co-occurrence in biological contexts to improve driver gene identification. DISHyper was retrained using the same data for consistent evaluation.

All models were evaluated using AUPRC, F1-score, and AUROC in pan-cancer and cancer-type-specific settings.

### Implementation details

All models were implemented using PyTorch and PyTorch Geometric (PyG). Training was carried out on a single NVIDIA V100 GPU with 32GB of memory. ONCOPLEX typically converged within 300 epochs. Unless otherwise specified, a learning rate of 0.001, a dropout rate of 0.5, and a hidden layer size of 128 were used.

## Supplementary Information

Supplementary Information 1.

Supplementary Information 2.

## Data Availability

Core features, including gene expression, mutation, and methylation profiles, were obtained from The Cancer Genome Atlas (TCGA) via the GDC portal (https://portal.gdc.cancer.gov/). The comprehensive feature set was downloaded from DORGE (https://doi.org/10.1126/sciadv.aba6784). Graphs were constructed using MSigDB pathway data (https://www.gsea-msigdb.org/). Cancer driver gene labels were collected from the Network of Cancer Genes v6.0, DigSee, COSMIC CGC v91, and IntOGen v2024.09.204. All processed data, including node features, graph structures, and gene labels, are available at: https://github.com/etab12/ONCOPLEX.
