# Supplementary material for: ONCOPLEX: an oncology-inspired hypergraph model integrating diverse biological knowledge for cancer driver gene prediction
Source: Sci Rep. 2026 Jan 13;16:5164. doi: 10.1038/s41598-026-36127-8 (PMC12881423; doi:10.1038/s41598-026-36127-8)
Supplement: Supplementary file 1 — Supplementary Information 1. [file 41598_2026_36127_MOESM1_ESM.pdf]

Supplementary Material

ONCOPLEX: An Oncology-Inspired Hypergraph Model Integrating Diverse Biological Knowledge for Cancer Driver Gene Prediction

Etab Mohammed Alotaibi[1], Omer S Alkhnbashi[2,3,*], and Van Dinh Tran[1,*]

[1]Information and Computer Science Department, King Fahd University of Petroleum and Minerals (KFUPM), Dhahran 34462, Saudi Arabia
[2]Center for Applied and Translational Genomics (CATG), Mohammed Bin Rashid University of Medicine and Health Sciences (MBRU), Dubai Health, Dubai, United Arab Emirates (UAE)
[3]College of Medicine - Mohammed Bin Rashid University of Medicine and Health Sciences (MBRU), Dubai Health, Dubai, United Arab Emirates (UAE)

1 Supplementary Methods

1.1 Datasets

Driver gene labels. We collect driver gene sets for both pan-cancer and cancer-specific driver genes from the following resources:

- **Network of Cancer Genes (NCG) v6.0**[1] http://ncg.kcl.ac.uk/download_file.php?file=cancergenes_list.txt
- **DigSee Database**[2] http://210.107.182.61/digseeOld/
- **COSMIC Cancer Gene Census (CGC) v91 and COSMIC Mutations in Census Genes**[3] https://cancer.sanger.ac.uk/cosmic/download
- **IntOGen tumor-specific driver lists v2024.09.20**[4] https://www.intogen.org/download

Gene sets used for biological validation. To further confirm our results, we assessed whether the newly predicted genes appeared in the following cancer gene databases:

- **OncoKB**[5] A manually curated resource of cancer genes annotated based on validated oncogenic effects. https://www.oncokb.org/cancerGenes
- **ONGene**[6] A collection of human oncogenes curated in the literature. http://ongene.bioinfo-minzhao.org/ongene_human.txt
- **CancerMine**[7] A text mining-based database of cancer genes extracted from the published literature.

1.2 Node features and normalization

We used three types of node features: incidence-based features, core omics features, and comprehensive features.

Incidence-based features: These features were derived solely from the hypergraph topology, without relying on any predefined biological node features. Each gene is represented by a binary matrix that captures its position within the pathway hypergraph. This setup ensures that the only source of information comes from the higher-order pathways connections.

Core omics features: These features were derived from multi-omics data collected in 16 types of cancer in the TCGA database[8]. For each type of cancer, we included three omics layers: Single-Nucleotide Variants (SNVs), gene expression, and DNA methylation. Following[9], the preprocessing steps are described below.

- **Single nucleotide variants:** We extracted the single-nucleotide variants from the TCGA Mutation Annotation Format (MAF) files. We computed the mutation frequency mf for each gene by normalizing the number of non-silent mutations by gene length:

$$mf = \frac{\sum m}{L},$$

where m is the number of mutations and L is the gene length. We excluded ultra-mutated samples to reduce noise due to genome instability.

- **RNA-Seq gene expression data:** TCGA provided RNA-Seq data as normalized FPKM counts. After batch correction, we computed the $\log_2$ fold change between tumor and matched normal samples:

$$\log_2 FC_g = \log_2\left(\frac{\text{FPKM}_g^{\text{tumor}}}{\text{FPKM}_g^{\text{normal}}}\right),$$

where $\text{FPKM}_g^{\text{tumor}}$ and $\text{FPKM}_g^{\text{normal}}$ represent normalized expression of gene g in tumor and normal samples, respectively.

- **DNA methylation:** We used beta values (ranging from 0 to 1) to compute differentially methylated regions. The logarithmic$_2$ fold change at the gene level was calculated as:

$$dmc_i = \frac{1}{|S_c|}\sum_{s\in S_c}\log_2\left(\frac{\beta_i^t}{\beta_i^n}\right),$$

where β_i^t and β_i^n are the beta values for the tumor and normal samples, respectively.

Each omics type generated a gene-by-sample matrix, and we combined the three matrices per cancer type to form a final multi-dimensional feature representation. In the pan-cancer setting, this resulted in a 48-dimensional feature vector per gene. For cancer-type-specific networks, we used only the corresponding omics features of that cancer, resulting in a 3-dimensional feature vector per gene.

Comprehensive features: The last set of node features, termed comprehensive features, is a 44-gene, epigenetic, and functional set of features collected from previous cancer driver prediction methods[10]. These features include biological properties at the gene level, such as histone modifications, evolutionary conservation, and gain/loss of function indicators. They are particularly useful in distinguishing oncogenes from tumor suppressor genes.

In the pan-cancer graph, we trained using all 44 comprehensive features. In cancer-specific settings, we integrated these comprehensive features with the cancer-specific omics features described above, enabling the model to jointly learn both cancer-type-specific and shared cross-cancer properties (see Table S12).

1.3 Hyperparameter optimization

A successful machine learning model depends on the selection of suitable hyperparameters, which makes careful tuning essential. We perform hyperparameter optimization using 5-fold cross-validation. The labeled data were divided into K folds, where (K-1) folds were used for training, and one fold was kept for testing. Within each training fold, an inner loop applied a grid search to explore combinations of hyperparameters and identify the optimal configuration based on validation performance. We tuned the following hyperparameters:

- Number of hidden units: [64, 128, 256]
- Number of layers: [2, 3, 4]
- Learning rate: [1e-3, 5e-4]
- Weight decay: [1e-3, 1e-4]
- Dropout: [0.25, 0.4, 0.5]
- Positive class weight: [0.2, 0.4, 0.45]

The **outer loop** was then used to evaluate the model with the best hyperparameter combination and report the average and standard deviation for the three metrics AUPRC, AUROC, and F1 score. This evaluation provides a robust and unbiased estimate of the performance of the model. We repeat the same procedures for the specific-cancer setting. In some types of cancer, the best set of hyperparameters is identical, while in other cases, a different set is optimal for that specific cancer.

2 Supplementary Tables

The following supplementary tables (**Tables S1–S14**) are provided as a single Excel file located in the same folder as this document, with each table presented in a separate worksheet.

Table S1: Comparison of the performance of ONCOPLEX and the baseline methods in the prediction task of the pan-cancer driver gene. The mean ± standard deviation of the AUPRC, AUROC, and F1-score is reported for each method in the evaluation runs.

Table S2: Comparison of the performance of ONCOPLEX and the baseline methods in 11 types of individual cancers. The mean ± standard deviation of the AUPRC, AUROC and F1-score are reported for each method and cancer type.

Table S3: Results of the numerical ranking of pan-cancer. The Hits value represents the average hits over five times for each method.

Table S4: Results of Pan-cancer ablation showing the impact of different configurations of node features on model performance. Values are reported as mean ± standard deviation in cross-validation folds for AUPRC, AUROC, and F1-score.

Table S5: AUPRC specific to the cancer-type in different configurations of features.

Table S6: Complete list of 30 newly predicted candidate genes for each cancer type. Genes are classified as common drivers or novel candidates not previously reported as cancer driver genes, and supporting evidence from established cancer-related databases is provided where available.

Table S7: New candidate genes identified in the top five ranked cancer types. For cancer types in which no novel genes appear among the top predictions, no entries are reported. The potential biological relevance of these genes was assessed through a comprehensive manual literature review.

Table S8: Results of the KEGG pathway enrichment analysis conducted on newly predicted genes in BRCA, HNSC, and STAD. Only significantly enriched pathways are reported.

Table S9: Significantly enriched Hallmark gene sets associated with newly predicted genes across cancer types, reported using the same statistical criteria as in **Table S8**.

Table S10: Overlap among the 100 newly predicted genes in different types of cancer. For each type of cancer, we evaluate how its predicted genes intersect with those of other cancers. As shown, some cancer types, such as BRCA, exhibit high levels of overlap with many others, suggesting shared molecular characteristics. In contrast, cancers such as ESCA show minimal overlap with most other types, except specific cases such as CESC, STAD, and LUAD, where moderate gene-level similarities are observed.

Table S11: The number of unique predicted genes identified for each cancer type. We analyze the genes that are exclusively predicted for a specific cancer type. Among all cancers, THCA and ESCA exhibit the highest number of unique genes, which can be attributed to their limited overlap in the pathways with other cancers. This reduced overlap of the pathway results in fewer shared genes with other types of cancer.

Table S12: Detailed description of the comprehensive feature set used in the second experiment to distinguish tumor suppressor genes from oncogene drivers between cancer types. The 44 features include mutation-related characteristics (e.g., missense and loss-of-function mutations), epigenetic signals such as DNA methylation and histone modifications, and an additional phenotype-based feature designed to improve predictive performance.

Table S13: Statistics of driver genes for each type of cancer based on the COSMIC and DigSEE databases. Passenger genes are shared across all types of cancer in the pan-cancer setting. The reported gene counts correspond to the genes included after hypergraph construction, with all genes successfully mapped to the network.

Table S14: Comparison of ONCOPLEX performance, measured by AUPRC, using core omics features with and without inclusion of copy number alteration (CNA) features. The results are presented for both pan-cancer and cancer-specific evaluation settings.

References

1. Repana, D. *et al.* The network of cancer genes (ncg): a comprehensive catalogue of known and candidate cancer genes from cancer sequencing screens. *Genome Biol.* **20**, DOI: https://doi.org/10.1186/s13059-018-1612-0 (2019).

2. Kim, J. *et al.* Digsee: Disease gene search engine with evidence sentences (version cancer). *Nucleic acids research* **41**, DOI: https://doi.org/10.1093/nar/gkt531 (2013).
3. Sondka, Z. *et al.* The cosmic cancer gene census: describing genetic dysfunction across all human cancers. *Nat. Rev. Cancer* **18**, DOI: 10.1038/s41568-018-0060-1 (2018).
4. Martínez-Jiménez, F. *et al.* A compendium of mutational cancer driver genes. *Nat. Rev. Cancer* **20**, 555–572, DOI: https://doi.org/10.1038/s41568-020-0290-x (2020).
5. Chakravarty, D. *et al.* OncoKB: A Precision Oncology Knowledge Base. *JCO Precis Oncol* 1–16, DOI: https://doi.org/10.1200/PO.17.00011 (2017).
6. Liu, Y., Sun, J. & Zhao, M. ONGene: A literature-based database for human oncogenes. *J. Genet. Genomics* **44**, 119–121, DOI: https://doi.org/10.1016/j.jgg.2016.12.004 (2017).
7. Lever, J., Zhao, E. Y., Grewal, J., Jones, M. R. & Jones, S. J. M. CancerMine: a literature-mined resource for drivers, oncogenes and tumor suppressors in cancer. *Nat Methods* **16**, 505–507, DOI: https://doi.org/10.1038/s41592-019-0422-y (2019).
8. Chang, K. *et al.* The cancer genome atlas pan-cancer analysis project. *Nat. Genet.* **45**, 1113–1120, DOI: https://doi.org/10.1038/ng.2764 (2013).
9. Schulte-Sasse, R., Budach, S., Hnisz, D. & Marsico, A. Integration of multiomics data with graph convolutional networks to identify new cancer genes and their associated molecular mechanisms. *Nat. Mach. Intell.* **3**, 1–14, DOI: https://doi.org/10.1038/s42256-021-00325-y (2021).
10. Lyu, J. *et al.* Dorge: Discovery of oncogenes and tumor suppressor genes using genetic and epigenetic features. *Sci. Adv.* **6**, eaba6784, DOI: https://doi.org/10.1126/sciadv.aba6784 (2020).
